# The Effect of Hypoxia and Hypoxia-Associated Pathways in the Regulation of Antitumor Response: Friends or Foes?

**DOI:** 10.3389/fimmu.2022.828875

**Published:** 2022-02-08

**Authors:** Raefa Abou Khouzam, Rania Faouzi Zaarour, Klaudia Brodaczewska, Bilal Azakir, Goutham Hassan Venkatesh, Jerome Thiery, Stéphane Terry, Salem Chouaib

**Affiliations:** ^1^ Thumbay Research Institute for Precision Medicine, Gulf Medical University, Ajman, United Arab Emirates; ^2^ Laboratory of Molecular Oncology and Innovative Therapies, Military Institute of Medicine, Warsaw, Poland; ^3^ Faculty of Medicine, Beirut Arab University, Beirut, Lebanon; ^4^ INSERM U1186, Gustave Roussy Cancer Campus, Université Paris-Saclay, Villejuif, France; ^5^ Faculty of Medicine, University Paris Sud, Le Kremlin Bicêtre, France; ^6^ Research Department, Inovarion, Paris, France

**Keywords:** hypoxia, tumor microenvironment, immune microenvironment, tumor plasticity, genetic instability, immunogenicity, hypoxia signature

## Abstract

Hypoxia is an environmental stressor that is instigated by low oxygen availability. It fuels the progression of solid tumors by driving tumor plasticity, heterogeneity, stemness and genomic instability. Hypoxia metabolically reprograms the tumor microenvironment (TME), adding insult to injury to the acidic, nutrient deprived and poorly vascularized conditions that act to dampen immune cell function. Through its impact on key cancer hallmarks and by creating a physical barrier conducive to tumor survival, hypoxia modulates tumor cell escape from the mounted immune response. The tumor cell-immune cell crosstalk in the context of a hypoxic TME tips the balance towards a cold and immunosuppressed microenvironment that is resistant to immune checkpoint inhibitors (ICI). Nonetheless, evidence is emerging that could make hypoxia an asset for improving response to ICI. Tackling the tumor immune contexture has taken on an *in silico*, digitalized approach with an increasing number of studies applying bioinformatics to deconvolute the cellular and non-cellular elements of the TME. Such approaches have additionally been combined with signature-based proxies of hypoxia to further dissect the turbulent hypoxia-immune relationship. In this review we will be highlighting the mechanisms by which hypoxia impacts immune cell functions and how that could translate to predicting response to immunotherapy in an era of machine learning and computational biology.

## Introduction

Solid tumors manifest in a microenvironment that harbors an array of cellular and non-cellular factors, cycling through various environmental pressures, which contribute to shaping a tumor’s immunological features (1). Hypoxia is an early event in tumor evolution that has been shown to both directly and indirectly impact this tumor immune microenvironment (TIME), with much of the evidence leaning towards an immunosuppressive influence (2–4). In addition, this condition of low oxygen is implicated in enabling tumor aggressiveness by providing tumor cells with a metabolic advantage (5) and by modulating autophagy (6). Hypoxia also promotes stemness and epithelial-mesenchymal transition (EMT) (7), genomic instability (8), and angiogenesis (9), thus contributing to all cancer hallmarks.

The repercussions of hypoxia in the TME occur through both hypoxia inducible factor (HIF)-dependent and independent mechanisms. HIF proteins are heterodimers composed of a constitutive β-subunit and an inducible α-subunit (HIF-1α, HIF-2α or HIF-3α). Under normal oxygen tension, HIF-1α and HIF-2α subunits are hydroxylated by prolyl hydroxylase (PHD), resulting in their subsequent ubiquitylation by the Von Hippel-Lindau tumor-suppressor protein (VHL), a component of an E3 ubiquitin ligase complex, and degradation by the proteasome (10). In hypoxic cells, HIF proteins are stabilized and in turn regulate the transcription of downstream genes, thereby modulating the microenvironmental stimuli within a tumor. The result is an acidic, nutrient deprived and immune-hostile microenvironment that is resistant to immunotherapy (2, 11). Indeed, limiting hypoxia in the TME in preclinical models has shown considerable improvement in the response to immune checkpoint inhibitors (ICI) (12, 13).

While there is emerging evidence of HIFs enhancing the activation status of immune cells in the TME (14–16), hypoxia is also known to mitigate their infiltration rate and function (3, 4). The TME comprises a slew of immune cell types, including those derived from the innate arm of the immune system, namely natural killer cells (NK) cells, macrophages and dendritic cells (DCs); as well as those belonging to the adaptive arm, including CD8+ effector T cells and CD4+ helper T cells. NK cells provide major histocompatibility complex (MHC)-unrestricted cytotoxicity against tumor cells (17). They also contribute to the sensitization of tumor cells to effector T cell killing by secreting the interferon, IFN-γ which acts on tumor cells upregulating their MHC and immunoproteasome expression (18, 19). With respect to macrophages, there is mounting evidence on the marked heterogeneity among populations resulting in a spectrum of macrophage subtypes with varying transcriptional sates (20, 21). This is contrary to the original binary model, wherein it is generally accepted that tumor associated macrophages (TAMs) display the so-called M2-like phenotype, which exhibits pro-tumorigenic features; while M1 macrophages have tumoricidal function and are classically activated (20). Dendritic cells are another subset of innate immune cells, classified as antigen presenting cells, that given the presence of co-stimulatory molecules and the correct cytokine environment, work to prime and activate T cells against tumor cells (22, 23). With respect to adaptive immune cells, the CD8+ effector T cells, or cytolytic T cells (CTLs) take the reigns as they are involved in direct tumor cell death through the induction of apoptosis and through cytokine secretion (24). The CD4+ T cells, on the other hand, exist as various subsets, among which the T helper 1 (Th1) subset is the most studied and is known to contribute antitumor activity by both direct killing and cytokine release; while regulatory T cells (Treg) and T helper 2 (Th2) cells constitute immunosuppressive subsets (24).

Hypoxia’s involvement in reprogramming the TME to one that is conducive to immune resistance has been evidenced multiple times, however exploring the intricacies of the hypoxia-immune cell relationship *in vivo* has been a challenge. The coupling of signatures reflecting the degree of a tumor’s hypoxic state with computational algorithms that can delineate its respective immune composition could uncover unexplored pathways and mechanisms of immune resistance. This is crucial given that an in depth understanding of the interplay between hypoxia-driven tumor cell remodeling and the immune contexture could aid in the betterment of patient response to immunotherapy. In this review, a link will be woven between the survival strategies taken up by tumor cells under hypoxic conditions and their impact on the immune microenvironment. In addition, recent findings from *in silico* analysis and the application of such tools to address hypoxia and the TIME will be discussed.

## Hypoxia Metabolically Reprograms the Tumor Microenvironment Excluding and Perturbing Immune Cell Function

The increased requirement for oxygen and nutrients within the hypoxic TME breeds a metabolic switch that works to nurture tumor survival, while posing as a functional barrier to the sustainability and activity of an anti-tumor immune response. When levels of molecular oxygen become too low to sustain mitochondrial adenosine triphosphate (ATP) production, a transition occurs from oxidative phosphorylation to glycolysis. This is supported by HIF-1α-induced upregulation of glucose transporters, which enhance the influx of glucose, that is in turn shuttled through the glycolytic pathway thanks to the HIF-1-mediated transactivation of key regulatory glycolytic enzymes, while inhibiting the tricarboxylic acid (TCA) cycle (5). As a byproduct of glycolysis, hypoxic cells concomitantly experience high levels of intracellular lactate and hydrogen ions. To overcome the eventual acidification of the cell, HIF-1α induces the expression of transporters and carbonic anhydrases to expel them (5, 25). The net effect is a glucose depleted and acidic TME with a pH as low as 5.8 to 6.5 (5, 26) and lactate concentrations reaching up to 30 mM, that is ten times higher than normal tissue (27). In such a TME, the anti-tumorigenic function of immune-activating cells is thwarted, while that of immunosuppressive cells is advocated.

Just like cancer cells, cytotoxic T cells also rely on glucose for aerobic glycolysis, which is itself necessary for their effector function (28); however, in the TME tumor cells outcompete T cells for glucose, thus inhibiting their antitumor activity (29). Glucose metabolism is further integral to the inflammatory phenotype in macrophages, the maturation and function of dendritic cells and NK cell activation (24, 30). Paradoxically, the immunosuppressive Tregs gain a metabolic advantage in a glucose deprived TME since they are less dependent on glucose as an energy source (31). Similarly, while Treg is resistant to the high lactate levels in the extracellular milieu (31), both NK cells and CD8+ T cells are encumbered by it. *In vitro* studies have shown that NK cell cytotoxicity and cytokine production are suppressed by high lactate and low pH levels (32, 33), as is CTL survival and function (4, 33). Lactate was shown to perturb DC maturation (34) and to drive Treg polarization from naïve T cells (35). Other immunosuppressive cells are likewise affected by the high lactate concentrations. The infiltration level of the T cell- and NK cell- suppressor, myeloid-derived suppressor cells (MDSC) is increased by tumor lactate (32). Tumor-derived lactate also induces the polarization of TAMs into the M2-like immunosuppressive phenotype (36) and treatment of a macrophage cell line with lactate promoted gain of M2-like features and downregulated the expression of cytokines, TNF-α and IL-12, secreted by M1 TAM (37). In addition, the acid-labile interferon, IFN-γ is rendered dysfunctional in this hostile TME, which in turn halts the maturation of anti-tumor M1 macrophages and promotes the differentiation of T helper cells to tumor promoting Th2 cells (38).

Apart from its impact on glucose metabolism, hypoxia interferes with amino acid and lipid metabolism, which are also essential for fueling cancer cell’s survival and modulating the immune contexture. The availability of the nonessential amino acid, glutamine, as well as other amino acids including tryptophan and arginine, which are vital for T cell function, can additionally be modulated by hypoxia. In particular, *in vitro* experiments showed that the deprivation of glucose or glutamine, which results in low α-ketoglutarate (α-KG), skewed CD4+ T cell differentiation in favor of immunosuppressive Treg cells (39). On the other hand, glutamine blockade *in vitro* and in tumor-bearing mice left CD8+ T cells metabolically intact and functional, while suppressing oxidative and glycolytic metabolism of cancer cells, leading to nutrient depletion, attenuated hypoxia, and acidosis in mice (40). This divergent response was attributed to the effector T cells using alternative sources as supplement for their long-lived, highly activated phenotype (24, 40). The opposite changes in cancer cells and effector T cells touch on a metabolic plasticity among the two that can be harnessed as an additional checkpoint for immunotherapy (24, 40). With respect to tryptophan, hypoxia has been shown to induce the expression of the rate limiting enzyme in its catabolism, indoleamine 2,3-dioxygenase 1 (IDO1). In macrophages, this resulted in suppressed T cell proliferation, coupled with enhanced expansion of immunosuppressive Tregs (41). Furthermore, IDO1 depletes tryptophan inducing an amino acid starvation response that promotes T cell anergy (42). While hypoxia was also shown to induce IDO-1 in DC (43), an opposite effect was reported in cancer cell lines of ovarian (44), cervical and glioblastoma (45) origins. Furthermore, the functional ortholog of IDO, TDO2 (tryptophan-2,3-dioxygenase), was found to be significantly downregulated in a HIF-1α dependent manner in glioblastoma cells exposed to hypoxia (46). TDO2 expressing cells in hypoxia were able to rescue T cell proliferation that is otherwise suppressed under normoxic conditions (46). The interplay between hypoxia and tryptophan metabolism is clearly riddled with controversial evidence, nonetheless, targeting IDO, as well as other players in the tryptophan catabolic pathway is being investigated in various clinical trials, alone or in combination with immune checkpoint inhibitors (47). In terms of arginine, it is metabolized rapidly by activated T cells and supplementing them with increased arginine levels was shown to enhance their anti-tumor activity *in vivo* (48). On the other hand, low arginine levels have been shown to suppress activating receptors of NK cells, like NKp30 and NKp46, to reduce the ability of NKs to produce IFN-γ and to impair their proliferation (24). Hypoxia has been shown to upregulate the expression of the two main enzymes in arginine metabolism, arginase 1 (ARG1) and the inducible nitric oxide synthase (iNOS), on MDSCs. This was in a HIF-1α-dependent manner and resulted in the differentiation of MDSCs into M2-like TAMs (49). MDSCs thereby compete with T cells for the utilization of this crucial amino acid, inhibiting T cell proliferation (50). Furthermore, *in vitro* coculture of macrophages with T cells in hypoxia promoted an increase in iNOS and ARG1 that resulted in T cell inhibition (51). Interestingly, another mechanism that leads to the induction of ARG1 on the surface of MDSCs may also be modulated by HIF1 and that is through the increased production of prostaglandin E (PGE). The inducible cyclooxygenase-2 (COX-2) in tumor cells leads to increased expression of PGE2, which has been shown to maintain the expression of ARG1 on the surface of MDSCs (52). COX-2 and the increase in PGE2 has also been shown to occur in a HIF-1α dependent manner, inhibiting the maturation of DC and enhancing the suppressive capacity of Tregs (53). Therefore, hypoxia could be compounding the depletion of arginine in the TME and the accompanying immunosuppression.

Along with its interference with nutrient uptake and their metabolism, hypoxia has been widely implicated in sending ATP metabolism into overdrive, which further feeds into an immunosuppressive outcome. In an inflammatory setting, extracellular ATP can be released by stressed and dying cells as well as activated monocytes and is involved in immune activation (54). The safety switch to halt the activated immune response and prevent damage of healthy tissue involves the phosphohydrolysis of extracellular ATP to adenosine; a process predominantly regulated by the two membrane-bound nucleotidases, CD39 and CD73 (54). Of interest, both ectonucleotidases are abundantly expressed in the TME and are additionally upregulated through a HIF-1α dependent mechanism in hypoxia (55). This is highly relevant in amplifying the immunosuppressive nature of the hypoxic TME, since adenosine possesses immune-dampening properties, repressing T cell effector function while stabilizing the suppressive function of Tregs (56). The immunosuppressive effects of extracellular adenosine have been well documented and are executed through the binding of this ligand to the Gs-protein-coupled receptors A2aR, expressed on the surface of monocytes, lymphocytes, NK cells and DC, as well as A2bR, which is most prominently expressed on DCs and macrophages (54, 56). Through these two purinergic receptors, adenosine triggers cyclic AMP (cAMP) accumulation. Within immune effector cells an increase in this intracellular signaling molecule results in an accumulation of an array of immunosuppressive molecules, including IL-10, TGF-β, PD-1 and CTLA-4, as well as the downregulation of key effector factors, such as IL-2, IFN-γ and perforin, which ordinarily participate in a pro-immune response (57). Hypoxia can further modulate adenosine levels through HIF-1α-dependent inhibition of adenosine kinase activity required to generate adenosine monophosphate (AMP), which in turn maximizes extracellular adenosine accumulation and depletes ATP levels in the cell (58). Indeed, the Hypoxia-Adenosine-Adenosine receptor axis represents various pharmacological targets and preclinical data support the rationale of combining A2aR blockade with hypoxia targeting strategies to reinvigorate the NK and T cell mediated anti-tumor immune response (59). Present data thereby suggests supplementing this combinational approach to current immunotherapeutic options to potentiate their efficacy (59).

## Hypoxia-Mediated Autophagy Plays a Double Role in the Immune Response

There is broad consensus that hypoxic stress in the tumor microenvironment activates autophagy mediated adaptation to low oxygen, however, autophagy outcome is still controversial and is observed as a double agent both promoting or suppressing tumor development commensurate to tumor type and staging which is strongly correlated to the therapeutic response. In fact, autophagy can mediate adaptive survival response to hypoxia (60–62) or a nonapoptotic programmed cell death called autophagy cell death (63, 64). Clinical data suggests a direct correlation between autophagy influx and tumor development. High Beclin-1 expression was linked with poor prognosis in advanced human nasopharyngeal carcinoma and colorectal adenocarcinoma samples (65, 66). Moreover, high autophagy turnover tumors were less sensitive to treatments in comparison with low autophagy turnover (67). As hypoxia-mediated autophagy induces more resistance to tumors in response to therapies than normoxic cells (68), a dual synergic treatment with autophagy inhibitors is suggested (69). During hypoxia, autophagy is induced through the activation of the HIF-1α which upregulated BCL2 Interacting Protein 3 (BNIP3) and BNIP3L, rendering Beclin-1 free to promote its interaction with VPS34 and the formation of autophagolysosomes (6). Alternatively, hypoxia induced autophagy can be mediated independently of HIF-1α through the Unfolded Protein Response (UPR) (70) or through the energy sensor AMP-activated protein kinase (AMPK) (64).

It has now been well documented that tumor lesions form, progress, and respond to therapy in the setting of a complicated interaction with the host immune system (71, 72). Data from genetically engineered mouse models demonstrated that autophagy influx influences the tumor cells as well as the immune cells in the TME (73, 74). Thus, autophagy machinery is suggested as a potential beneficial pharmacological and genetic target to mitigate anti-tumor immune responses (75–79) with some successful preclinical data. Notably, immune cells’ activation, differentiation and proliferation can be modulated by autophagy, which mediates promotion or inhibition of tumor development. Under hypoxic condition, as in tumors, immune cells experience hypoxia and have to adjust their metabolic needs and may do so through autophagy machinery which plays a plethora of action of immune cells to regulate anti-tumor immune reaction. CD8+ cells differentiation to cytotoxic T lymphocytes (80), their infiltrating and stemness preservation (81), T cells differentiation to Th cells, iNKT cells survival, differentiation and proliferation (82), DCs and B cells development (82), Treg cells survival, stability and immune tolerance (83), monocytes differentiation into macrophages and polarization and the number of macrophages as well (84), MDSCs growth and the establishment of T cell memory (85–87) are enhanced by autophagy. However, autophagy negatively regulates neutrophils development and induces their degradation (88).

Similarly to its dual impact on tumors development, autophagy can be observed as immune-simulator or immune-suppressor in the context of immune-mediated tumor elimination (88). It even becomes more complex when tumors are submitted to hypoxic stress. Understanding the contribution of hypoxia-induced autophagy in immune response to tumors is instrumental for better shaping therapeutic strategies. Thus, many studies showed the implication of multiple signaling pathways in hypoxia inducing autophagy to downregulate immune responses. Hypoxia-induced autophagy can attenuate NK cells anti-tumor activity. Loss of HIF-1α in NK cells blocks tumor growth (89) and hypoxia upregulation of HIF-1α in NK cells is dependent on PI3K/mTOR signaling pathway activation in response to cytokine receptor gamma chain, reducing NK cells tumor suppressive function (90). Moreover, hypoxia can modify the transcriptome of NK cells, regulating their immunoactivity and influencing their migration which may profoundly influence their infiltrating capacity in tumor tissues (91). Once X-irradiated, NK cells became more resistant and maintain killing capacity under hypoxic conditions (92). Recently, data demonstrated a strong correlation between attenuated NK cell cytotoxicity and a decreasing level of phosphorylated STAT3 and ERK through protein tyrosine phosphatase SHP-1 (Src homology region 2 domain-containing phosphatase-1) activation (93). Both STAT3 and ERK phosphorylation is increased in pre-activated hypoxic NK cells which restores their proliferation under hypoxic conditions (94). In contrast, hypoxia-induced autophagy attenuates CTL-mediated tumor degradation by activating the Src Kinase, which phosphorylates STAT3 in a HIF-1α dependent manner (95, 96). Simultaneously, HIF-1α induces autophagy thought the Beclin-1- BNIP3- Bcl-2 axis, resulting in the degradation of the SQSTM1/p62 protein responsible for the degradation of p-STAT3 leading to its accumulation in cells (97, 98) and preventing CTL attacks. Concomitantly, STAT3 expression promotes HIF-1α expression and modulates hypoxia-induced EMT in esophageal squamous cell cancer (99). Moreover, hypoxia-induced autophagy degrades NK-derived Granzyme B in tumor cells through the autophagy sensor Inositol 1,4,5-Trisphosphate Receptor Type 1 (ITPR1), thus impairing NK-mediated tumor cell degradation (100). Furthermore, hypoxia-induced autophagy has been associated with destabilization of the immune synapse between the NK and the tumor cells through decreasing the level of connexin 43 leading to impairment of NK killing efficacy (101, 102).

## Hypoxia-Driven Tumor Plasticity and Heterogeneity Incite Immune Escape

### Epithelial to Mesenchymal Transition (EMT) and Stemness in the TME

Optimum oxygen levels are essential to maintain tissue homeostasis. When oxygen sensing mechanisms or when oxygen levels decrease, a cascade of molecular events escalates a multitude of responses. Stabilization of HIF-1α ensues molecular changes that initiate EMT. EMT is characterized by an increase in cell migration, invasion, production of extracellular matrix (ECM) and resistance to apoptosis (103). Specific transcription factors are activated by HIF-1α to mediate EMT phenotypes, these include SNAIL SLUG, TWIST1, ZEB1, SIP1/ZEB2 (104). Additional pathways that have been shown to be involved in the hypoxia-mediated EMT include TGF-β, Wnt/β-catenin, hedgehog and Notch (104).

The process of EMT in cancer cells endows them with stem cell features. These newly formed cancer stem cells (CSCs) result in a heterogeneous cancer cell population. CSCs just like normal tissue stem cells can adapt a quiescent cellular state, characterized by a cell cycle arrest with reduced metabolic activities (105). In addition, CSCs have the capacity to self-renew and differentiate, as such they are credited for tumor growth, invasive growth, and metastasis at distal sites (106). The quiescent state of CSCs contributes to their resistance to therapeutic drugs (105). However, targeting CSCs requires an understanding of the several developmental signaling pathways that function to mediate and maintain their self-renewal and differentiation, these include TGF-β/BMP, Notch, Wnt, Hedgehog, FGF, and IGF (107). These signaling pathways are interconnected and overlapping and are present at crossroads that feedback into the hypoxia axis: TGF-β/SMAD3 pathway can be activated by HIF1 and at the same time it results in the stabilization of HIF1 by suppressing PHD levels (104). Notch interacts with HIF-1α to turn on expression of genes important in maintaining the undifferentiated cell state (108). In addition, Notch signaling regulates SNAI1 as well as hypoxia-induced cell motility and invasion (108). Wnt/β-catenin signaling has a pro-EMT effect in cells under hypoxia (109). Furthermore, HIF‐1α knockdown abolishes hedgehog pathway activation (110). Finally, FGF induces HIF-1α expression (111).

Identifying cancer stem cells relies on the expression of unique markers on their cell surface. Depending on the cancer type these include EpCAM, ALDHA1, Lgr5, CD13, CD24, CD26, CD47, CD49f/Integrin alpha 6, CD66c, CD90, CD166, CD271, CD105, CD44, CD133, CD117/c-kit, CD138, CD151 and CD166 (112). CD44 and CD133 are the most widely used markers. CD44 is a transmembrane glycoprotein that is expressed in solid and hematological cancers, it mediates stromal interaction and has different activation states upon binding to its ligand hyaluronan (HA). In this context, CD44 has been shown to be active on cancer cells and not in normal cells (113). CD44 constitutes a potential marker to enhance targeted therapy, indeed in breast cancer CD44-doxorubicin conjugated aptamers inhibited selective cell proliferation of CD44 expressing cells (114). CD133 is a transmembrane glycoprotein expressed in several tumors. A variety of promising immunotherapeutic strategies have been developed to target CD133 expressing cells (115).

### Impact of Plasticity and Heterogeneity on Tumor Immune Escape

Hypoxia-driven tumor plasticity and heterogeneity may have substantial impact on immunosuppression and cancer immune evasion (116). The pioneer study of Ye and colleagues previously revealed that hypoxia-induced EMT of hepatocellular carcinoma cells can promote an immunosuppressive TME by stimulating the release of the CCL20, leading to the production of IDO by monocyte-derived macrophages, which in turn suppressed T cell proliferation and promoted the expansion of immunosuppressive regulatory T cells (41). In fact, there are many known potential factors such as TGF-β contributing to hypoxia-driven tumor immune escape (3, 7) evoking features of cancer stem cells and tumor epithelial-mesenchymal plasticity. We previously showed that the stemness-associated transcription factor NANOG is induced by hypoxic stress; not only conferring stemness properties to carcinoma cells, but it also increases TGF-β expression and secretion, thereby promoting infiltration of immunosuppressive cells in the murine B16 melanoma model (117). We also showed that hypoxic stress can promote EMT programs enhancing immune evasion of NSCLC carcinoma cells (118). In the human IGR-Heu model, the tumor population was found to be highly heterogeneous following hypoxic stress, with an important fraction of cells conserving marked epithelial features. The mesenchymal cancer clones were found to have increased intrinsic TGF-β pathway activity and increased capacity to resist attacks by immune cytotoxic effector cells compared to the more epithelial clones, as reflected by reduced cancer cell susceptibility to CTL and NK cell-mediated lysis. To note, heterogeneity also exists within the mesenchymal clones. For instance, the expression of the receptor tyrosine kinase AXL could mark cancer clones with pronounced immune evasion capacity in association with reduced expression of ICAM1, ULBP1, and MHC class I levels in cells (119). It is interesting to consider that AXL expression can be upregulated by many intrinsic as well as extrinsic factors including hypoxia (120, 121). Published data have been unclear across different cancer systems and models. Research is still needed to decrypt the regulatory events controlling the expression of AXL and more generally of the TAM (Tyro3 Axl Mertk) family receptors. On the other hand, AXL activity has been shown to support a hypoxic state in carcinoma cells by stabilizing tumoral HIF-1α through cooperation with HER2 (122). In a murine Her2+ breast cancer model, this cooperative event greatly contributed to shaping an immunosuppressed TME, while Axl targeting led to improving anti-Pd-1 treatment efficacy.

Work by Zhang and colleagues revealed that HIF-1α can stimulate CD47 expression in breast cancer cells gaining stemness features, which also serves as a mechanism to evade phagocytosis by innate immune cells such as macrophages (123). The CD47 SIRP interaction hampers the “eat me signal” on macrophages impairing phagocytosis. Another study found that the CD47 gene is a direct target of EMT-associated transcription factors SNAI1 and ZEB1 (124). Moreover, CD47 and PD-L1 can act synergistically to sustain resistance and immunosuppression (123). Interestingly, carcinoma cells with stemness features certainly exhibit immunogenicity profiles that differ from well-differentiated carcinoma cells with consequences on tumor immunogenicity, neo-antigen expression and the anti-tumor immune response (125). Complex interactions between the different contingents should also be highlighted. For instance, Faget et al. showed that neutrophils in lung tumors alter angiogenesis and immunotherapy efficacy by promoting tumor hypoxia and partial EMT of carcinomas as events of a vicious cycle maintaining an immunosuppressed pro-tumoral microenvironment (126).

Thus, several studies have demonstrated the role of hypoxia mediated-EMT and plasticity on tumor immune escape, although with variability in terms of the mechanisms and cell types involved. It will be important to better integrate the intratumor heterogeneity parameter in future investigations. Another important challenge will be to translate this information into the clinic with safe effective strategies.

### Hypoxia-Dependent Modulation of Cancer Cell Glycosylation as a Mediator of Immune Escape

Another aspect of cancer cells that is modified by hypoxia and plays a role in the modulation of the immune response is glycosylation. Addition of glycans is a posttranslational modification that regulates the activity of as many as 50% of human genes, making it one of the most important regulators of gene expression. In tumors, abnormal glycosylation gives rise to a glycol-profile that perpetuates key cancer hallmarks of proliferation, EMT, angiogenesis, invasion, and metastasis (127, 128). Moreover, immune response itself is highly controlled by glycosylation [as reviewed in (129)]. Altered glycan residues on cancer cell surface, usually related to increased or unusual sugar components, give rise to tumor-associated carbohydrate antigens (TACAs) which are weakly immunogenic, and thus may serve as an immune escape strategy (130–132). Glycosyltransferases and glycosidases that add and remove sugar residues, respectively, are highly modulated by hypoxia in a tumor-dependent fashion (127, 128). In addition, hypoxia contributes to increasing specific structures involved in tumor invasion and immune escape (127, 128). In particular, the highly hypoxic muscle-invasive bladder cancer (MIBC) overexpresses the cancer-associated carbohydrate antigen sialyl-Tn (STn), which has been reported to be at least in part due to a HIF-1α-dependent cell survival strategy that favors cell migration and invasion (133). Recently, through the application of glycoproteomics in bladder cancer, the same group demonstrated cell surface expression of an ordinarily intracellular protein, homer homolog 3 (HOMER3), carrying short-chain O-glycans that are characteristic of membrane proteins (134). They further reported that under glucose deprivation and hypoxic conditions, HOMER3 contributed to the tumor cell’s invasive capacity. Of interest, cell-surface expression of this protein was associated with significantly worse survival in MIBC patients. Furthermore, while HOMER3 expression was not cancer-specific, STn and HOMER3 did not co-express in healthy tissue, suggesting that HOMER3-STn could be a tumor-specific biomarker that can be used to target the more aggressive cancer cell populations residing in the hypoxic TME (134). Similarly, some cancer-specific glycoconjugates, like N-glycolyl (NeuGc) GM3 gangliosides, are promising therapeutic targets, as their increased expression is characteristic in tumors and almost not present in healthy human tissues (135). Gangliosides are glycosphingolipids containing sialic acid residues and their expression can be induced by hypoxia (136). While the exact mechanism is unknown, hypoxia has been shown to induce the sialic acid transporter, sialin (137). In addition, despite humans lacking the functional enzyme responsible for N-glycolyl (NeuGc) GM3 synthesis, hypoxia upregulates the succinate dehydrogenase subunit B (SDHB) of the mitochondrial respiratory complex II, which is hypothesized to provide the deleted iron/sulfur catalytic domain of the enzyme, restoring its functionality (138). Gangliosides, and in particular GM3(NeuGc), have been showed to play a key role in suppressing the antitumor immune response and therefore serve as neoantigens that can be targeted by immunotherapy (135). Indeed, given that they can also induce antibody responses, several clinical trials are ongoing to use them as anti-cancer vaccine antigens, as has recently been reviewed (135, 139).

## Role of Hypoxia in Angiogenic Switch and Pro-Angiogenic Milieu Modulation of Immune Response

Initially, a growing tumor remains avascularised and relies on the diffusion of oxygen and nutrients from surrounding tissues (140) or reprograms metabolically to survive hostile, O_2_ and nutrient limited environment (as discussed above). However, upon progression a phenotypic change in cancer cells occurs, termed angiogenic switch when balance of secreted factors moves from anti- towards proangiogenic. This event causes dramatic change in the tumor milieu that primarily induces angiogenesis (141). Previously mentioned HIF-1α stabilization in response to hypoxia activates in cancer cells not only adaptation to low pO_2_ but also production of one of the most potent proangiogenic factors, vascular endothelial growth factor (VEGF). Other proangiogenic factors secreted by cancer cells include fibroblast growth factor (FGF) family, interleukin-8 (IL-8), epidermal growth factor (EGF) and platelet-derived growth factor (PDGF) (142). In response to such milieu [and, also hypoxia itself, reviewed elsewhere (143)], in order to supply the growing tumor, endothelial cells (ECs) from surrounding tissues are activated to form new vessels. However, due to dysregulation of proangiogenic response in cancer cells, the forming vessels are disturbed, as a consequence of pathological angiogenesis, contributing even more to cancer progression (144).

### Pathological Endothelium and Consequences for Immune Homing

Cancer-associated, pathological vessels are characterized by leakiness and disturbed shape causing uneven vascularization of the tumor mass. Cellular and ECM composition of cancer-associated vessels are altered, which limits their barrier functions causing uncontrolled transport of nutrients, oxygen, and drugs. Consequently, due to abnormalities, newly formed vessels are not able to restore physiological level of oxygen within the tumor, and hypoxia sets in. Impaired perfusion and increased interstitial pressure of cancer vessels were shown to negatively affect leukocyte trafficking (145). Additionally, composition of immune homing receptors is altered in cancer-associated endothelial cells, which affects the infiltration of the tumor with leukocytes. In a steady state, ECs remain quiescent, regulate blood flow and barrier functions of the endothelium, however upon activation, for example in response to inflammation, expression of adhesion molecules changes, allowing leukocyte trafficking into the organ. On one hand, pro-inflammatory molecules, like TNF-α or IL-1α, which can be secreted by cancer cells, activate ECs. On the other, pro-angiogenic factors (VEGF, bFGF) were shown to reduce the amount of adhesion molecules on ECs (146). These dual effects are reflected in leukocyte trafficking through the tumor endothelium. It was shown that vessels present in the tumors lose P-selectin that limits the infiltration of leukocytes, making tumors inaccessible for the immune response (147). High VEGF levels were linked to lower levels of ICAM1 and T cell infiltration (145). This is affected by anti-cancer treatment, as ipilimumab plus bevacizumab could restore ICAM/VCAM expression on ECs, enhancing infiltration of cytotoxic lymphocytes (148). On the other hand, levels of E-selectin were increased in tumoral vessels in breast cancer and were also present in surrounding inflamed adipose tissue vasculature (149). Expression of this adhesion molecule allowed monocyte infiltration; however, these monocytes could be TAMs as their presence predicted poor survival. In pancreatic cancer, upregulation of adhesion proteins on the endothelium, including E-selectin, MAdCAM-1 and VCAM-1, allowed increased infiltration of Tregs (150). E-selectin was shown to favorably promote infiltration of non-protective Th2- polarized cells (151). Immune infiltrate can also be shaped due to the production of chemokines by tumor endothelial cells (TECs). ECs in the TME were characterized by downregulation of immuno-attractant molecules (CCL2, CCL18, IL-6) (152), additionally further limiting the leukocyte infiltration. At the same time, TECs were described to possess a specific secretory profile, including IL-4, -13, -6, -8, and TNF-α, which can modulate immune responses (153). Another way ECs can affect immune cells is by directly interacting with them, for example through PD-L1. It was shown that endothelial PD-L1 is increased in several cancers in comparison to healthy tissue, which coincided with lower infiltration of T cells and dominance of Tregs (154).

Therefore, alteration of adhesion molecule patterns and levels of secreted factors in TECs can mediate selective infiltration of immunosuppressive leukocytes promoting tumor growth or make the tumor impenetrable for protective immune cells (so called “cold”, uninflamed tumors). This points to the role of ECs as contributors to shaping the immunosuppressive TME.

### Immune-Modulating Role of VEGF and Other Pro-Angiogenic Factors

Apart from the modulatory role on ECs, proangiogenic factors were shown to affect immune cells’ function. It was observed that VEGF can directly expand Tregs, recruit MDSC and inhibit DC maturation (155). VEGFR is selectively present on Tregs and not effector T cells, which explains homing of immunosuppressive cells into proangiogenic TME (156). It is also a known factor promoting Th2 responses that are usually not protective in cancer. Additionally, VEGF induces expression of immunosuppressive PD-1 on T lymphocytes (157). Another proangiogenic factor with strong immunomodulatory potential is FGF (158). It was shown to polarize macrophages into M2 subtype (159) and expand MDSCs (160). Interestingly, anti-FGF treatment caused broader T-cell receptor repertoire, probably due to increased cancer cells apoptosis (161) that shows an additional aspect that can be altered by proangiogenic and immunomodulatory molecules. Similarly, however less studied, activities were reported for PDGF. This growth factor is an important regulator of angiogenesis, especially during development (162), and tends to increase during EMT in cancer cells (163). It can inhibit maturation of DCs and induce IL-10 producing T cells with regulatory phenotype (164). A strongly angiogenic chemokine, IL-8 (165), affects several immune cells, mostly by promoting their adhesion to the endothelium and subsequent migration towards inflamed tissue. However, it was observed that IL-8 mediates recruitment into the tumor of MDSCs and N2, pro-tumoral neutrophils (166), pointing to the potential immunomodulating action of this chemokine.

To sum up, angiogenic switch and consequently pathological angiogenesis on several levels affect immune response. As factors shaping TME, they contribute to induction of immunosuppression and/or allow the tumor to remain immunoevasive, both by not alleviating hypoxia and maintaining a pro-angiogenic and immunomodulatory milieu.

## The Hidden Potential of Hypoxia-Modulated Genetic Heterogeneity in Evoking an Immune Response

In the tumor microenvironment, hypoxia is often associated with genomic instability through downregulation of DNA repair processes and replication signaling mechanisms. Although the DNA damage sensing and signaling mechanisms are on high-alert for recognition of plausible DNA damage under acute/chronic hypoxia, the DNA repair pathways such as homologous recombination, Non-Homologous End Joining (NHEJ) and mismatch repair, are downregulated (8). The effect of hypoxia on DNA repair pathways and related genes has been reviewed elsewhere (167–169). In contrast, chronic hypoxia/anoxia for longer durations can induce replication stress due to downregulation of ribonucleotide reductase and depletion of deoxyribonucleotides (170). The downregulation of these processes contributes to genetic heterogeneity in tumors through induction of chromosomal instability, point mutations, and genome-doubling events (8). Hypoxia induced structural changes (large deletions, copy number aberrations, duplications, and truncations) are substantially higher than single nucleotide alterations, according to a recent study examining the pan-cancer data sets (171). Hypoxia is associated with increased mutational load and hypoxia associated early mutations occur in key driver genes like *BCL2*, *TP53*, *MYC*, *PTEN* and *VHL* (171). Although driver mutations contribute to clonal development of tumors, branching mutations are the major cause of intratumor genetic heterogeneity and play a key role in drug resistance (172). In a clinical setting, irrespective of the tumor type, branched evolution remains the norm and influence the tumor’s evolutionary trajectory (173). The contribution of hypoxia to branched evolution of tumors can be extrapolated from a study done by Gerlinger and coworkers analyzing the effect of *VHL* driver mutations in clear cell renal cell carcinomas (ccRCC). *VHL* mutations are seen in 80% of the ccRCC and contribute to constant pseudohypoxia phenotype (174, 175). Using multi-region sequencing and phylogenetic analysis, the study revealed that the inactivation of the *VHL* gene through mutations/methylation were a founder event in the trunk of the phylogenetic trees (176) and showed a heterogeneity in genomic landscape among the subclones with wide-ranging clinical outcome. *In vitro* experimental studies have shown that hypoxia induces a panoply of single nucleotide variations and contributes to microevolution of tumors. However, in a clinical setting, it is noted that chromosomal instability is a major contributor of tumors heterogeneity and a major determinant of clinical outcome in cancers (177). Hypoxia exerts selection pressure to accelerate the adaptation of more competent chromosomally unstable tumor clones in several ways (178). Hypoxia triggers the selection of mutant clones (for example, *TP53*-mutated tumors) by allowing them to evade apoptotic mechanisms (179). A hypoxic microenvironment promotes cell competition and metastases by HIF-1α mediated epithelial-mesenchymal transition (178). Hypoxia drives the immune-escape of tumors by inducing the expression of immune checkpoint inhibitors and controlling the antigen presenting mechanisms (180). Genome-doubling events associated with hypoxia have been found *in vitro* in melanoma cells with an increase in levels of tetraploid cells, however, such events in clinical samples are not well understood (181).

Inactivation of DNA repair pathways can lead to significant increase in tumor mutational burden (182, 183). The contribution of DNA repair and replication processes to genomic instability under hypoxic conditions is clearly evident from the defective homologous recombination and defective mismatch repair related mutational signatures (171). Single base substitution signatures and insertion and deletion signature analysis reveals that high-hypoxia is associated with clonal mutations in tumors rather than subclonal mutations (171). Furthermore, hypoxia is associated with increase in APOBEC activity and cyclic hypoxia induced replication stress provides single stranded DNA substrates for APOBEC mediated mutagenesis in breast, lung, and colorectal cancers (184).

Increased neoantigen load renders the tumor immunogenic with increased infiltration of lymphocytes leading to better clinical response to ICI in non-small cell lung cancer, melanoma, colorectal adenocarcinoma (185). In a breast cancer model, recent research from our group found that tumor hypoxia increased tumor mutational load and potential neoantigens (186). Using publicly available datasets, Bhandari and coworkers revealed that high-hypoxia is associated with increased TMB at pan-cancer level (171). However, clinical evidence on hypoxia-induced TMB and neoantigen burden is lacking. On the contrary, tumor hypoxia leads to an ‘immune-cold’ environment. Hypoxic tumor microenvironment is associated with immune evasion through expression of immune checkpoints (programmed death ligand -1), downregulation of type-I interferon signaling, shedding of antigen presenting molecules (MHC class I), enrichment of immunosuppressive cytokines and aggregation of immune suppressive cells (MDSC and Tregs) in the TME (187). In this regard, recent attempts to target hypoxic cells selectively with hypoxia activated prodrugs have yielded encouraging results with a significant antitumor response to immune checkpoint blockade. Jayaprakash and coworkers, using transgenic adenocarcinoma of the mouse prostate (TRAMP-C2), demonstrated that hypoxia targeting through Evofosfamide restored the T cell infiltration within the tumor and enhanced the response to immune checkpoint blockade (12). A study by Lequeux and coworkers investigated the inhibition of HIF-1α activity on cytotoxic immune cell infiltration into B16-F10 melanoma, and found an increase in infiltration of NK and CD8+ effector T cells and a significantly increased response to anti-PD-1 blockade (13). A comprehensive understanding of hypoxia induced mutational burden, neoantigen load will be crucial for enhancing the immunotherapy response in ICI resistant tumors.

## Unraveling the Hypoxia-Immune Contexture *In Silico*: The Hits and the Misses

The methods utilized thus far to study the immune contexture and degree of hypoxia in tumors have mainly done so separately. Regarding tumor infiltrating immune populations, imaging techniques including immunohistochemistry (IHC) and fluorescence microscopy, as well as cytometry-based methods, using cell/population-specific antibodies have been the standard approach (188). For hypoxia, in addition to IHC which is used to check hypoxia induced proteins (like CAIX and GLUT1), various imaging techniques, such as positron emission tomography (PET), oxygen-enhanced (OE) magnetic resonance imaging (MRI), as well as blood oxygen level dependent (BOLD) and tissue oxygen level dependent (TOLD) MRI, have been utilized (9, 106). The focus of this section, however, is the uprise of *in silico* approaches to simultaneously navigate both the immune and hypoxic aspects of the TME.

In the last decade, the application of hypoxia gene signatures to reflect the degree of tumor hypoxia has taken the literature by storm with published signatures covering almost every solid tumor type (9, 106). In addition to that, there has been an escalating number of papers focused not only on designating the hypoxic state of a tumor, but also interrogating the immune populations and immune activation status of that tumor depending on its hypoxic phenotype (189–197). The process generally entails first deriving a hypoxia signature in the cancer type of interest, which often takes the route of narrowing down a list of hypoxia-related differentially expressed genes (DEGs) between normal and tumor tissue, then determining which genes are correlated with patient prognosis, be it overall survival or disease-free survival. The top genes and the factor by which they influence survival are then put together in a formula to calculate the risk score. Each sample is then allocated to the high-risk or low-risk group depending on their expression of the signature genes and whether their score is greater or less than the median risk score of the entire cohort. The score in this case is not only reflective of the hypoxic state of the tumor but is also associated with worse patient prognosis. An alternate strategy has also been used to group patients into high and low hypoxia groups based on their hypoxia score (197). The hypoxia score is calculated according to their expression levels of the hypoxia signature genes alone, without incorporating a risk parameter. Here again, the distribution is based on the variation from the median expression of the signature genes, and higher score is associated with worse survival. In either case, the next step has been to apply different tools or immune signatures to compare the two groups to make conclusions on the immune microenvironment in the context of hypoxia.

Several computational tools exist that rely on a tumor’s bulk transcriptomic data to enumerate its existing immune populations (188, 198). These tools employ both a selected statistical framework as well as a base signature matrix or gene set representing the immune cell types of interest to deduce the tumor’s respective immune phenotype (188, 199). The statistical framework is a variation of one of two primary algorithms, enrichment, or deconvolution. Gene set enrichment gives a semiquantitative score describing the enrichment of a cell type of interest in a sample based on the ranking of cell-type specific marker genes compared to all other genes present (198). A variation of that algorithm is single-sample GSEA (ssGSEA), in which the enrichment score is computed to represent the coordinately upregulated or downregulated genes within a single sample (198, 200). On the other hand, deconvolution algorithms consider the transcriptome profile of a heterogenous sample as a linear mixture of gene expression levels of distinct cell types. The unknown cell fraction of interest can then be estimated by determining the weighted contribution of each gene to a signature matrix that includes the cell-type specific expression profiles (198). In this way, tools based on the deconvolution algorithm give quantitative estimates of relative cell fractions; however, given that in a heterogeneous sample cell types having higher amounts of total mRNA will have a stronger contribution to the mixture, such cell types may be overestimated (198).

An important determinant for the effective estimation of the immune cell populations is the quality and accuracy of the gene set or base signature matrix being incorporated by the computational tool (201, 202). For example, Estimation of STromal and Immune cells in MAlignant Tumours using Expression data (ESTIMATE) uses a gene set derived from the overlap between gene expression profiles (GEPs) of normal hematopoietic samples and genes associated with the quantity of immune cells infiltrating tumor tissue (203). This gene set constitutes the immune signature and is used to give a tumor sample an immune score. The tool also has a gene set representing the stromal signature and uses that to give the same sample a stromal score. The combination of the two scores indicates the ESTIMATE score, or tumor purity.

With respect to deconvolution-based tools, the first base signature matrices to be used were derived from microarray data conducted on FACS-derived subsets of cells originating from peripheral blood mononuclear cells (PBMCs) of healthy individuals, or *in vitro* stimulated and differentiated cells (204, 205). These result in suboptimal coverage of cellular phenotypes in complex tissues and prevent the discovery of possible new cellular states, as well as gene expression profiles that are cell-type specific (206). Furthermore, tools based on such base matrices, are only compatible with microarray derived gene expression profile of a tumor sample. Such tools, include Cell-type Identification By Estimating Relative Subsets Of RNA Transcripts (CIBERSORT), which can be applied with a leukocyte signature matrix representing 22 immune cells to deconvolute and resolve the relative fractions of these cells in complex tissue (205). To overcome the stated limitations, an upgraded computational framework of CIBERSORT, termed CIBERSORTx, has been formulated using cell type-specific reference profiles derived from single cell RNA sequencing, allowing cross-platform normalization and *in silico* cell purification (206). Starting form RNA expression profiles of intact whole tissue samples, the cell-type-specific GEPs and abundance of each cell type can then be accurately inferred (206). Indeed, this tool is heralded as a digital cytometer that negates the need for physical dissociation, living material or antibodies, yet manages to give a detailed portrait of tissue components from bulk RNA admixtures (206).

In addition to ESTIMATE, CIBERSORT and CIBERSORTx, other commonly used tools include Microenvironment Cell Populations (MCP)-counter, which computes an abundance estimate of eight different immune cell types and two stromal cells (fibroblasts and endothelial cells) (207); as well as the webtool Tumor IMmune Estimation Resource (TIMER), which provides the proportions of six immune cells, CD4+ and CD8+ T cells, B cells, DCs, macrophages, and neutrophils in the tissue of 23 tumor types from The Cancer Genome Atlas (TCGA) (208). One more tool of interest is ImmuCellAI (Immune Cell Abundance Identifier) that uses signatures to give abundance estimates on 24 immune cells including 18 subtypes of T cells, as well as B cells, DCs, monocytes, NK cells, macrophages, and neutrophils. Both RNA-Seq and microarray expression data are compatible with this tool (209). A final tool worth mentioning here is TIDE, which stands for Tumor Immune Dysfunction and Exclusion. This tool goes beyond determining the immune status of a tumor and potential immune escape. It was developed to use gene expression profiles of cancer samples to predict response to ICI by reporting on both immune and stromal cellular elements, a tissue agnostic interferon gamma signature, as well as the enrichment scores of ICI biomarkers, microsatellite instability and PD-L1, among others (210).

**Table 1** represents select studies that have used one or a combination of the computational approaches to annotate the tumor immune microenvironment and merged that with a hypoxia gene signature to distinguish the more hypoxic from the less hypoxic tumors. As evident from the table, every signature consists of its own set of genes, even if it was derived from the same tumor type, with minimal overlap with other signatures. The conclusions of most papers underscore the immunosuppressive power of hypoxia in bladder cancer (189, 213), breast cancer (214), colorectal cancer (CRC) (191, 216, 217), head and neck squamous cell carcinoma (218), hepatocellular carcinoma (HCC) (221–223), lung cancer (195, 225, 226), melanoma (196), oral squamous cell carcinoma (227), osteosarcoma (229), ovarian carcinoma (230) and pancreatic ductal adenocarcinoma (PDAC) (197, 231). In the case of bladder cancer, two other studies determined the presence of high infiltration of immune cells in the high-risk group and found positive correlations between immune score and the risk score (190, 212). The authors also showed that despite the presence of both tumor-promoting and tumor-antagonizing immune cells, the risk score was positively correlated with immune checkpoints. One study even went on to report a potentially enhanced response of the high-risk group to immunotherapy (190). With respect to CRC, the only contradictory study included a single cohort and only focused on GSEA and the ESTIMATE score, not considering immune populations (215). In terms of HCC, one study reported a higher immune score in the high-risk group (219) as well as a significant infiltration of immune cells in this group which also showed enhanced predicted response to ICI (220). In a study that integrated 11 independent HCC cohorts, not a single immune cell population could be significantly differentiated in a consistent manner between the low-risk and high-risk groups, highlighting the complexity of the factors determining immune cell infiltration (194). It is difficult to make any conclusion on the findings in renal cell carcinoma, as all three studies included a single cohort respectively and reported distinct findings (232–234) (**Table 1**).

**Table 1:** Studies applying computational tools to investigate the immune landscape of tumors classified based on hypoxia signatures.

s.n	Cancer	Hypoxia Signature	Cohort Number^‡^	Immune Investigation Method	High-Risk Group (Hypoxia-High)	Low-Risk Group (Hypoxia-Low)	Reference
**1**	ACC^*^	3 genes (*CCNA2*, *COL5A1*, *EFNA3*)	1	CIBERSORT	Resting NK cell	Activated NK cell	(211)
**2**	**BLCA**	8 genes (*AKAP12*, *ALDOB*, *CASP6*, *DTNA*, *HS3ST1*, *JUN*, *KDELR3*, *STC1*)	3	CIBERSORT	M0 and M1 macrophages		(189)
**3**	**BLCA^#^**	4 genes (*ANXA2*, *COL5A1*, *GALK1*, *HS3ST1*)	1	ESTIMATE; ssGSEA	Immune and stromal scores positively correlated with risk score		(212)
					Activated CD4 T cell, activated CD8 T cell, central memory CD8 T cell, effector memory CD8 T cell, gamma delta T cell, follicular helper T cell, Th1, Th2, aDC, pDC, activated B cell, immature B cell, memory B cell, NK cell, NK T cell, Treg, macrophage, MDSC, mast cell, monocyte, neutrophil, eosinophil	Th17, CD56bright NK cell	
**4**	**BLCA^#^**	16 genes (*AKAP12*, *ANKZF1*, *CASP6*, *CCNG2*, *GALK1*, *GAPDH*, *HDLBP*, *HEXA*, *HS3ST1*, *SDC4*, *SLC2A1*, *SLC2A3*, *SRPX*, *STC1*, *VEGFA*, *WISP2*)	2	ssGSEA	CD8 T cell, NK cell, DC, Th1		(190)
					Risk score positively related to T cell inflamed score and enrichment scores of immunotherapy-positive gene signatures		
**5**	**BLCA^#^**	7 genes (*ALDOB*, *EGFR*, *FOXO3*, *GPC1*, *SDC4*, *SLC2A3*, *VEGFA*)	1	CIBERSORT	Resting mast cell, neutrophil, resting CD4 memory T cell	Follicular helper T cell, CD8 T cell, plasma cell	(213)
**6**	**BC**	13 genes (*ADM*, *ALDOA*, *CDKN3*, *LDHA*, *MIF*, *MRPS17*, *NDRG1*, *P4HA1*, *PGAM1*, *SLC2A1*, *TPI1*, *TUBB6*, *VEGFA*)	1	ImmuCellAI	nTreg cell, iTreg cell	CD8 T cell, CD4 T cell	(214)
**7**	**CRC**	5 genes (*ARL4C*, *CARS2*, *PSMD12*, *PTTG1IP*, *SEC61G*)	1	GSEA; ESTIMATE	Enriched immune pathways		(215)
					Positively correlated with immune score and stromal score		
**8**	**CRC**	12 genes (*CASP6*, *CYB5R3*, *DTX3L*, *FAM117B*, *IRF1*, *MBTD1*, *MINPP1*, *ORAI3*, *TNFAIP8*, *TRAF3*, *PRELID2*, *ZBTB44*)	2	ESTIMATE; CIBERSORT	Treg, M2 macrophage	Higher immune and stromal scores; CD4 T cell, M1 macrophage	(216)
**9**	**CRC**	4 genes (*ALDOB*, *ALDOC*, *GPC1*, *SLC2A3*)	2	CIBERSORT	M0 macrophage		(217)
**10**	**CRC**	356 genes	4	CIBERSORT	M0 and M2 macrophages	CD8 T cell, resting NK, resting CD4 memory T cell	(191)
**11**	**GC^#^**	2 genes (*EFNA3*, *SERPINE1*)	2	ESTIMATE; ssGSEA	Higher immune and stromal scores; Treg, macrophage, neutrophil, mast cell		(192)
**12**	**Glioma^#^**	5 genes (*GAPDH*, *HK2*, *JUN*, *LDHA*, *VEGFA*)	2	CIBERSORT	Resting CD4 memory T cell, Treg, resting NK cell, M0 macrophage, neutrophil		(193)
**13**	**HNSCC**	24 genes (*AMPD3*, *BHLHE40*, *COL5A1*, *CP*, *CSRP2*, *CXCR4*, *DDIT4*, *DUSP1*, *ERRFI1*, *F3*, *GPC4*, *HS3ST1*, *IL6*, *ISG20*, *MAFF*, *PGM2*, *PIM1*, *PLAC8*, *PPP1R3C*, *S100A4*, *SDC2*, *SELENBP1*, *SERPINE1*, *SRPX*)	1	CIBERSORT	Activated DC, M0 macrophage, eosinophil, activated mast cell, resting NK cell, resting CD4 memory T cell	Memory B cell, CD8 T cell, resting mast cell, Treg, follicular helper T cell, activated CD4 memory T cell, gamma delta T cell, plasma cell, activated NK cell	(218)
**14**	**HCC**	4 genes (*ENO1*, *GAPDH*, *LDHA*, *SLC2A1*)	1	ESTIMATE CIBERSORT	Higher immune score; Treg, M0 macrophage, neutrophil	Activated NK cell, M1 macrophage, resting mast cell	(219)
**15**	**HCC^#^**	24 genes (*ACOT7*, *ADM*, *ALDOA*, *ANGPTL4*, *BNC1*, *CA9*, *CDKN3*, *COL4A6*, *ENO1*, *FOSL1*, *GNAI1*, *LDHA*, *MIF*, *MRPS17*, *NDRG1*, *P4HA1*, *PGAM1*, *PGK1*, *SDC1*, *SLC16A1*, *SLC2A1*, *TPI1*, *TUBB6*, *VEGFA*)	1 meta-cohort (from 3 datasets)	MCP-counter; ssGSEA; TIDE	CD4 T cell, activated CD8 T cell, iDC, aDC, CD56bright and CD56dim NK cell, gamma delta T cell, immature B cell, macrophage, mast cell, MDSC, NK T cell, pDC, follicular helper T cell, Th1, Th2; Three times higher response to ICI	Eosinophil, neutrophil, Treg, Th17	(220)
**16**	**HCC^#^**	21 genes (*ADM*, *BNIP3*, *BNIP3L*, *CA9*, *EGLN3*, *GDF15*, *GYS1*, *HCAR3*, *HILPDA*, *HK2*, *INSIG2*, *JUN*, *KDM3A*, *PFKFB4*, *PLIN2*, *PTPRH*, *SLC2A3*, *SMAD3*, *SPAG4*, *TMEM45A*, *WSB1*)	11	ESTIMATE; CIBERSORT	3 cohorts: higher stromal score; 6 cohorts: higher immune score; 6 cohorts: activated CD4 memory T cell; 5 cohorts: activated mast cell; 5 cohorts: M0 macrophage	6 cohorts: resting CD4 memory T cell; 5 cohorts: resting mast cell; 3 cohorts: NK cell	(194)
**17**	**HCC^#^**	3 genes (*CDCA8*, *PDSS1*, *SLC7A11*)	1	CIBERSORT	M0 macrophage, memory B cell, follicular helper T cell		(221)
**18**	**HCC**	10 genes (*APEX1*, *ATR*, *CTSA*, *DNAJC5*, *ENO1*, *EPO*, *HMOX1*, *LDHA*, *NDRG1*, *PER1*)	1	CIBERSORT	M0 macrophage, Treg, neutrophil, eosinophil	Resting mast cell, resting CD4 memory cell, M1 macrophage, monocyte	(222)
**19**	**HCC**	4 genes (*DCN*, *DDIT4*, *NDRG1*, *PRKCA*)	2	ssGSEA		Activated B cell, activated CD8 T cell, effector memory CD8 T cell, Treg, Th1, CD56bright NK cell, NK cell, NK T cell, eosinophil, macrophage, mast cell, MDSC, monocyte, pDC	(223)
**20**	NSCLC^*^	11 genes (*AMPD3*, *DDX11*, *FANCI*, *HIF-3α*, *IDE*, *LRP8*, *NOLC1*, *PAIP1*, *PDCD2*, *PSMF1*, *SNAPC5*)	1	ESTIMATE; ssGSEA		Higher immune score; DCs, aDCs, iDCs, pDCs, HLA, B cell, mast cell, neutrophil, T helper cell, T cell co-inhibition, T cell co-stimulation, TILs, Type II IFN response	(224)
**21**	**NSCLC**	4 genes (*ANGPTL4*, *PFKP*, *SLC2A1*, *XPNPEP1*)	2	CIBERSORT	Activated CD4 memory T cell, resting NK cell, M0 and M1 macrophages	Memory B cell, resting CD4 memory T cell, monocyte, resting DC, resting mast cell	(195)
**22**	**NSCLC**	18 genes (*ADM*, *BIK*, *DDIT3*, *ENO1*, *EPAS1*, *FGF3*, *GAPDH*, *MIF*, *NFKB1*, *PFKP*, *PGK1*, *PLAUR*, *SPP1*, *STC1*, *TEK*, *TFRC*, *TGFA*, *XRCC6*)	1	ssGSEA	Activated CD4 T cell, CD56bright NK cell, memory B cell, Th2	Activated B cell, activated CD8 T cell, central memory CD4 T cell, effector memory CD8 T cell, eosinophil, immature B cell, iDC, pDCs, macrophage, mast cell, MDSC, monocyte, NK cell, neutrophil, follicular helper T cell, Th1, Th17	(225)
**23**	**NSCLC^#^**	7 lncRNAs (AC010980.2, AC022784.1, AC079949.2, AC090001.1, AL161431.1, LINC00707, LINC00941)	1	CIBERSORT	Neutrophil, M0 and M2 macrophages	Monocyte	(226)
**24**	**SKCM**	11 genes (*CP*, *DPYSL4*, *EGFR*, *FBP1*, *FOXO3*, *IGFBP1*, *ISG20*, *KIF5A*, *PPARGC1A*, *S100A4*, *SDC3*)	2	CIBERSORT	Treg, mast cell; 1 cohort: resting CD4 memory T cell, monocyte	Activated CD4 memory T cell, M1 macrophage; 1 cohort: CD8 T cell, plasma cell	(196)
**25**	**OSCC^#^**	4 genes (*ALDOA*, *P4HA1*, *PGK1*, *VEGFA*)	1	CIBERSORT	M0 macrophage, mast cell	Naïve B cell, CD8 T cell, follicular helper T cell, Treg, neutrophil	(227)
**26**	OS^*^	2 genes (*P4HA1*, *DCN*)	2	ssGSEA		DC, pDC, macrophage, neutrophil, TIL	(228)
**27**	**OS**	4 genes (*EFNA1*, *P4HA1*, *STC2*, *MAFF*)	2	CIBERSORT	Resting CD4 memory T cell		(229)
**28**	**OVC^#^**	9 genes (*ALOX5AP*, *ANXA1*, *IGFBP2*, *LAG3*, *PLK3*, *SLC1A1*, *SREBF1*, *SREBF2*, *TGFB1*)	1	CIBERSORT; TIMER	Activated CD4 memory T cell, gamma-delta T cell, activated NK, neutrophil, M1 and M2 macrophages	Resting CD4 memory T cell, follicular helper T cell, Treg, aDC, resting mast cell; Higher MHC and antigen presenting molecules	(230)
**29**	**PDAC^#^**	8 genes (*DDIT4*, *LDHA*, *MXI1*, *NDRG1*, *P4HA1*, *PGK1*, *SLC2A1*, *VEGFA*)	2	CIBERSORTx	M0 macrophage	CD8 T cell; Higher immune score and cytolytic index^§^	(197)
**30**	**PDAC**	4 genes (*ENO3*, *LDHA*, *PGK1*, *PGM1*)	2	CIBERSORT	M2 macrophage, resting NK cell	CD8 T cell, naive B cell	(231)
**31**	**RCC**	9 lnRNA (AC002070.1, AC008760.2, AC084876.1, AC147651.1, FOXD2-AS1, ITPR1-DT, LINC00944, LINC01615, LINC02027)	1	CIBERSORT	Plasma cell, follicular helper T cell, Treg	M2 macrophage, resting DC, resting mast cell	(232)
**32**	**RCC**	4 lnRNA (AC026462.3, COMETT, EMX2OS, HAGLR)	1	TIMER; ESTIMATE	B cell, CD4 T cell, CD8 T cell, DC, macrophage, neutrophil positively correlated with risk score		(233)
					Higher immune and stromal scores		
**33**	**RCC^#^**	8 genes (*BCL2*, *KDELR3*, *KLF6*, *PCK1*, *PLAUR*, *PPARGC1A*, *RORA*, *WSB1*)	1	CIBERSORT	Treg, CD8 T cell, follicular helper T cell, plasma cell, M0 macrophage, activated NK cell	Resting CD4 memory, monocyte, M1 macrophage, resting mast cell, resting NK cell	(234)

^‡^Number of independent patient cohorts analyzed with indicated method to investigate immune tumor microenvironment.

^#^Studies reporting higher immune checkpoint inhibitors or immunosuppressive cytokines or both in High-risk group.

^*^Studies reporting higher immune checkpoint inhibitors or immunosuppressive cytokines or both in Low-risk group.

^§^Immune score calculated based on an eighteen gene tumor inflammation signature. The cytolytic index calculated based on the geometric mean of the GZMA (granzyme A) and PRF1 (perforin-1) produced by activated cytolytic CD8+ T cells (197).

s.n, serial number; ACC, adrenocortical carcinoma; BLCA, bladder urothelial carcinoma; BC, breast cancer; CRC, colorectal cancer; GC, gastric cancer; HNSCC, head and neck squamous cell carcinoma; HCC, hepatocellular carcinoma; NSCLC, non-small cell lung cancer; SKCM, skin cutaneous melanoma; OSCC, oral squamous cell carcinoma; OS, osteosarcoma; OVC, ovarian carcinoma; PDAC, pancreatic ductal adenocarcinoma; RCC, renal cell carcinoma; lnRNA, long non-coding RNA; CIBERSORT, Cell-type Identification By Estimating Relative Subsets Of RNA Transcripts; ESTIMATE, Estimation of STromal and Immune cells in MAlignant Tumours using Expression data; GSEA, gene set enrichment analysis; ssGSEA, single-sample GSEA; MCP-counter, Microenvironment Cell Populations-counter; TIMER, Tumor IMmune Estimation Resource; ImmuCellAI, Immune Cell Abundance Identifier; DC, dendritic cell; aDC, activated DC; iDC, immature DC; pDC, plasmacytoid DC; Th1, type 1 T helper cell; Th2, type 2 T helper cell; Th17, T helper 17 cell; Treg, regulatory T cell; iTreg, induced Treg; nTreg, natural Treg; HLA, human leukocyte antigen; TIL, tumor infiltrating leukocytes; IFN, interferon; MHC, major histocompatibility complex.

It is clear that to resolve the discrepancies identified for even the same tumor type, a validated hypoxia gene signature should be utilized to score the tumor and the *in silico* analysis of the immune contexture should be done using signatures that reflect the immune state of the specific cancer type. The tools utilized thus far apply gene expression profiles obtained from immune cells of healthy PBMCs or tissue, meanwhile based on the complexity of mechanisms governing the activation state of immune cells, a cancer-specific approach would provide a more accurate representation of the immune contexture. This would ultimately enhance the quality of the findings being generated from these tools, making them more consistent and having higher accuracy. Furthermore, one downside remains with the inability of such tools to give information about the localization of the reported immune populations in the tumor mass. This spatial dimension is intercalated with the global functional state of the immune response and is now being investigated with advanced techniques, including single cell transcriptomics using slide sequencing (235), as well as multiplex immunofluorescence imaging using the CODEX^®^ System (236). Therefore, despite the simplicity and ease of use of current computational tools, they should not be used as a standalone analysis platform to conclude on the immune activation state of a tumor.

## Discussion

To date, clinical benefit from cancer immunotherapy has been limited to a minority of patients. Achieving benefit in the majority of patients necessitates a wholistic understanding of anti-tumor response mechanisms and both the cell-intrinsic and extrinsic molecules involved in primary, adaptive, as well as acquired resistance to immunotherapy. In this regard, it has become clear that the TME is likely to play a crucial role in cancer response to treatment. In fact, the growth and progression of cancer cells depend not only on their malignant potential, but also on the multidirectional interactions of cellular and metabolic components of tumor microenvironment. It is widely admitted that novel and continuously evolving pathological entities arise as a result of the interactions among tumor cells and stromal cells during cancer progression.

As previously reported, many cellular, molecular, and metabolic elements of the TME are emerging as attractive targets for therapeutic approaches (47, 59, 115, 122). In this respect, the existence of hypoxia in solid tumors is associated, not only with tumor invasion and metastasis, but also with a heightened risk of treatment failure and patient mortality and is currently attracting significant interest. Accumulating evidence indicates that hypoxia plays a key role in promoting the acquisition of tumor resistance to various antitumor immune effectors (26). Tumor hypoxia allows tumor cells to escape CTL- and NK -mediated killing through in part the activation of autophagy (97, 237, 238) and modulates the composition and function of the immune infiltrate (97, 237, 238). Hypoxic zones in tumors have also been reported to attract immunosuppressive cells such as MDSCs, tumor-associated macrophages and regulatory T cells. In addition, the association of hypoxia with cancer stemness in the tumor microenvironment of different cancer types is widely admitted. Therefore, controlling hypoxic stress to avoid tumor resistance and to reshape the hypoxic immunosuppressive TME in order to improve cancer immunotherapy remains a relevant challenge. Developing pharmacological agents to modulate HIF-1α signaling pathway is still attracting significant interest in the field of oncoimmunology. Several sub-types of drugs have been reported to inhibit HIF-1α activity including inhibitors of HIF-1α/HIF-1β dimerization (for example, acriflavine) (239, 240). Very recently, we demonstrated that suppression of the transcriptional activity of HIF-1α resulted in an increased infiltration of NK cells and CD8+ T cells in the tumor microenvironment of melanoma (13). Hypoxia could therefore be a potential immunometabolic checkpoint with prognostic value by regulating the TME and affecting the interaction between tumor cells and immune cells.

It is now well established that high expression of clonal tumor neoantigens correlates with an upregulation of lymphocyte infiltration within a tumor, enhanced patient survival and a prolonged response to immunotherapy. Recently, others and we have demonstrated that hypoxia interferes with genetic instability by inducing DNA damages, inducing DNA repair alteration (186) and presumably the emergence of tumor neoantigens. While the main predictive biomarkers for immunotherapy involve microsatellite instability/defective mismatch repair (MSI/dMMR), and tumor mutational burden, based on our previous reports and those of other teams, tumor hypoxia should be also be exploited as a potential biomarker to predict immunotherapy outcomes.

A deeper understanding of the role of hypoxia in killer cell induction and migration, immune suppression and EMT could enable the creation of more highly refined, innovative and integrative immunotherapies, targeting tumor plasticity and heterogeneity and aiding in overcoming the inherent constraints of currently applied anticancer therapies. In addition to the known hypoxic signatures reported, we believe that the design of minimally- or even non-invasive techniques able to predict treatment efficacy and tumor recurrence through algorithm-based modeling of network dynamics and by generating models based on artificial intelligence, or through the integration of “omics”, must be considered in the field of oncoimmunology.

## Author Contributions

All authors listed have made a substantial, direct, and intellectual contribution to the work, and approved it for publication.

## Funding

This work was funded by the Gulf Medical University.

## Conflict of Interest

The authors declare that the research was conducted in the absence of any commercial or financial relationships that could be construed as a potential conflict of interest.

## Publisher’s Note

All claims expressed in this article are solely those of the authors and do not necessarily represent those of their affiliated organizations, or those of the publisher, the editors and the reviewers. Any product that may be evaluated in this article, or claim that may be made by its manufacturer, is not guaranteed or endorsed by the publisher.
